# Purification and Biochemical Characterization of TsMS 3 and TsMS 4: Neuropeptide-Degrading Metallopeptidases in the *Tityus serrulatus* Venom

**DOI:** 10.3390/toxins11040194

**Published:** 2019-03-31

**Authors:** Daniela Cajado-Carvalho, Cristiane Castilho Fernandes da Silva, Roberto Tadashi Kodama, Douglas Oscar Ceolin Mariano, Daniel Carvalho Pimenta, Bruno Duzzi, Alexandre Kazuo Kuniyoshi, Fernanda Vieira Portaro

**Affiliations:** 1 Immunochemistry Laboratory, Butantan Institute, São Paulo SP 05503-900, Brazil; cristiane.silva@butantan.gov.br (C.C.F.d.S.); roberto.kodama@butantan.gov.br (R.T.K.); bruno.duzzi@butantan.gov.br (B.D.); alexandre.kuniyoshi@butantan.gov.br (A.K.K.); 2 Biochemistry and Biophysics Laboratory, Butantan Institute, São Paulo SP 05503-900, Brazil; douglas.mariano@butantan.gov.br (D.O.C.M.); dcpimenta@butantan.gov.br (D.C.P.)

**Keywords:** *Tityus serrulatus*, metalloserrulases, proteases, purification, biochemical characterization, neuropeptides

## Abstract

Although omics studies have indicated presence of proteases on the *Tityus serrulatus* venom (TsV), little is known about the function of these molecules. The TsV contains metalloproteases that cleave a series of human neuropeptides, including the dynorphin A (1-13) and the members of neuropeptide Y family. Aiming to isolate the proteases responsible for this activity, the metalloserrulase 3 and 4 (TsMS 3 and TsMS 4) were purified after two chromatographic steps and identified by mass spectrometry analysis. The biochemical parameters (pH, temperature and cation effects) were determined for both proteases, and the catalytic parameters (*K_m_*, k*_cat_*, cleavage sites) of TsMS 4 over fluorescent substrate were obtained. The metalloserrulases have a high preference for cleaving neuropeptides but presented different primary specificities. For example, the Leu-enkephalin released from dynorphin A (1-13) hydrolysis was exclusively performed by TsMS 3. Neutralization assays using Butantan Institute antivenoms show that both metalloserrulases were well blocked. Although TsMS 3 and TsMS 4 were previously described through cDNA library studies using the venom gland, this is the first time that both these toxins were purified. Thus, this study represents a step further in understanding the mechanism of scorpion venom metalloproteases, which may act as possible neuropeptidases in the envenomation process.

## 1. Introduction

Scorpion accidents have been the main cause of human envenomation by animals in Brazil since 2007 [1]. These occurrences are associated with the easy adaptation of scorpions to urban centers, which offer shelter, food availability, and the absence of natural predators [2]. In particular, the *Tityus serrulatus* is considered the main Brazilian species of medical importance, as it is responsible for a greater number of severe envenomation cases when compared with other scorpion species [3,4]. Accidents are especially lethal when they occur with children under five years of age and the elderly, becoming a relevant public health problem in Brazil. Thus, there is interest from the scientific community regarding the study of this species and its venom components.

Animal venoms are a mixture of toxins with diverse biological effects, which could be used as a source for bioactive molecules. Among these, peptidases are often found in animal venoms, especially in snakes [5,6]. For example, snake venoms from the Viperidae family are known to be comprised of metallo and serinoproteases [7]. Proteolytic enzymes are the main toxins in most of these venoms and accidents symptoms are frequently associated to the activities of these molecules, mainly severe haemostatic disturbances, like consumptive coagulopathy and local or systemic hemorrhage [8,9].

Scorpion venoms, on the other hand, are better known and characterized by their neurotoxic activity [10]. However, little is known about their proteolytic components and their role during the envenomation process. This could be partially explained by the lack of information about the effects of scorpion venom peptidases, or even due to the difficulty of obtaining ideal amounts of these venoms for the isolation of such molecules. Fortunately, “omics” techniques (proteomic and transcriptomic) have recently helped the study of peptidases in these arachnids [11,12,13,14]. Notably, transcriptomic studies have shown that proteases are the most abundant transcripts in the Brazilian scorpions from the *Tityus* genus, representing 48%, 38% and 33% of the venom glands transcripts of *Tityus obscurus*, *T. bahiensis* and *T. serrulatus* respectively [14,15]. Also, cDNA analysis of the *T. serrulatus* venom gland using a primer for the M13 metalloproteinase family, revealed the presence of clones of ten peptidases, which were named “metalloserrulases”. Nine putative sequences (TsMS 1 to 9) have the signature of the metzincins family, a group of metalloproteases including matrix metalloproteinases and ADAM proteases. Differently, the last one, TsMS 10, is classified as a member of the gluzincin family, group which includes enzymes like the angiotensin I-converting enzyme, the endothelin I-converting enzyme and the neprilysin [16]. These classifications are based on a conserved sequence of catalytic sites of zinc metallopeptidases, since metzincins have an extended zinc-binding sequence (HEXXHXXGXXH), in addition to a Met-turn methionine that helps to coordinate the metal ion; and gluzincins which have a shorter catalytic site (HEXXH), in addition to a glutamate helix below the active-site helix [17]. Finally, metalloserrulases 1 and 2 (TsMS 1 and TsMS 2) showed the highest percentage of similarity with the primary structure of antarease, which could indicate similar activities; while metalloserrulase 4 (TsMS 4) had the lowest similarity with this scorpion venom protease, suggesting diversity of functions [16].

Antarease was the first protease purified from the *Tityus serrulatus* venom (TsV), which was characterized by the hydrolysis of proteins involved in the transport of vesicle to the cell membrane, the VAMPs [18]. In addition, Zornetta et al. (2017) showed that the active recombinant antarease caused neuroparalysis at the neuromuscular junction of rats and flies (*D. melanogaster*) by the cleavage of proteins on the surface of the pre-synaptic membrane, thus preventing neuroexocytosis. Moreover, this observation was attributed to proteolytic activity, since the inactive enzymatic mutant protein did not achieve the same effect [19]. Ortiz et al. (2014) showed, by transcriptome analysis, the existence of antarease molecules in venoms of other species of scorpions from Central America (*T. fasciolatus*, *T. pachyurus*), North America (*Centruroides noxius*) and Asia (*Mesobuthus*), indicating conservation of these enzymes in the Buthidae scorpion family, regardless of their geographic origin [20]. The second metallopeptidase purified and characterized from TsV was an Angiotensin-Converting Enzyme-like (ACE) peptidase [21], and its ability to release angiotensin II from angiotensin I might contribute to hypertension, which is a symptom that is commonly described on patients envenomed by the *T. serrulatus* scorpion. Interestingly, the ACE-like sequence was also found in transcripts from scorpion venom glands of the Buthidae family [11,14,15,21], which suggest conservation of this molecule on scorpion venoms during their evolution.

Besides the transcriptomic analyzes, peptidomic studies have detected the activity of endopeptidases and exopeptidases over endogenous venom-peptides, being considered important post-translational agents for the formation of toxins [22].

Regarding functional activities, studies using the total venom showed the enzymatic activity of metallopeptidases on venoms of Brazilian *Tityus* scorpions (*T. serrulatus*, *T. bahiensis* and *T. stigmurus*), which is able to inactivate the neuropeptide dynorphin A (YGGFLRRIRPKLK), releasing Leu-enkephalin (YGGFL), another active human neuropeptide [23]. In another study, endopeptidases and exopeptidases from the *T. serrulatus* venom were able to cleave human peptides in vitro, with members of the neuropeptide Y family being the best hydrolyzed substrates [24].

The observation that the venom of *Tityus serrulatus* contains metallopeptidases acting on essential human neuropeptides—dynorphin A (1-13), neuropeptide Y, peptide YY and pancreatic polypeptide—was the motivation for the development of the present study, since the neurotoxic syndromes are the main symptoms presented by victims of accidents and these molecules are broadly distributed in the body, being found mainly in the adrenal gland, peripheral nervous system and also in immune cells [25,26,27,28]. Thus, here we describe the isolation and biochemical characterization of metalloserrulases 3 and 4, TsMS 3 and TsMS 4, from the *T. serrulatus* venom, and their in vitro proteolytic activities on human neuropeptides.

## 2. Results

### 2.1. Isolation of TsMS 3 and TsMS 4

**Figure 1:**
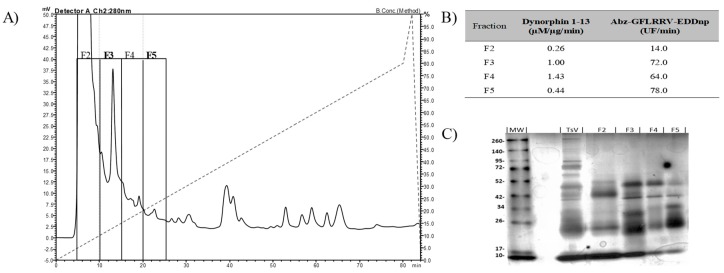
(**A**) Fractionation of *Tityus serrulatus* venom by anion-exchange chromatography on a PA-DEAE column in HPLC system. (**B**) Specific activity of fractions F2–F5 to substrates hydrolysis. The substrates used were dynorphin A 1-13, hydrolysis of which was visualized on the C-18 RP-HPLC system, and the fluorescent substrate Abz-GFLRRV-EDDnp, hydrolysis of which was monitored using fluorimeter Victor 3 (Perkin Elmer). (**C**) The SDS-PAGE (12%) of active fractions eluted from the PA-DEAE. Lane MW: molecular mass markers; lane TsV: *T. serrulatus* venom.

Metalloserrulases 3 and 4 were purified following two chromatographic steps, as summarized in Table 1, which aimed to maintain the proteolytic activity upon dynorphin A (1-13)—Dyn A (1-13)—and its fluorescent homologue Abz-GFLRRV-EDDnp. First, the solubilized venom was fractioned by anion exchange chromatography and 4 active fractions were obtained (Figure 1, panel A). Fractions F3 and F5 stood out for having high hydrolysis rates on both substrates tested (Figure 1, panel B). However, a different hydrolysis pattern over dynorphin A 1-13 was observed. According to the mass spectrometry results, while fraction F3 hydrolyzed the substrate Dyn A (1-13) in the same manner as the total venom, releasing Leu-enkephalin (YGGFL) (Supplementary Figure S1, panel A), the fraction F5 activity presented only a single cleavage point, with no formation of Leu-enkephalin (only YGGFLR ↓ RIRPKLK) (Supplementary Figure S1, panel B). The SDS-PAGE showed that the F3 and F5 fractions have similar profiles, but with different intensities and some unique components in each fraction (Figure 1, panel C). Subfractions F3 and F5 also hydrolyzed neuropeptides from the neuropeptide Y family (data not shown). Furthermore, all hydrolyzes over fluorescent and natural substrates were inhibited in the presence of the EDTA chelating agent, indicating metalloproteinases activities (Table 1).

**Figure 2:**
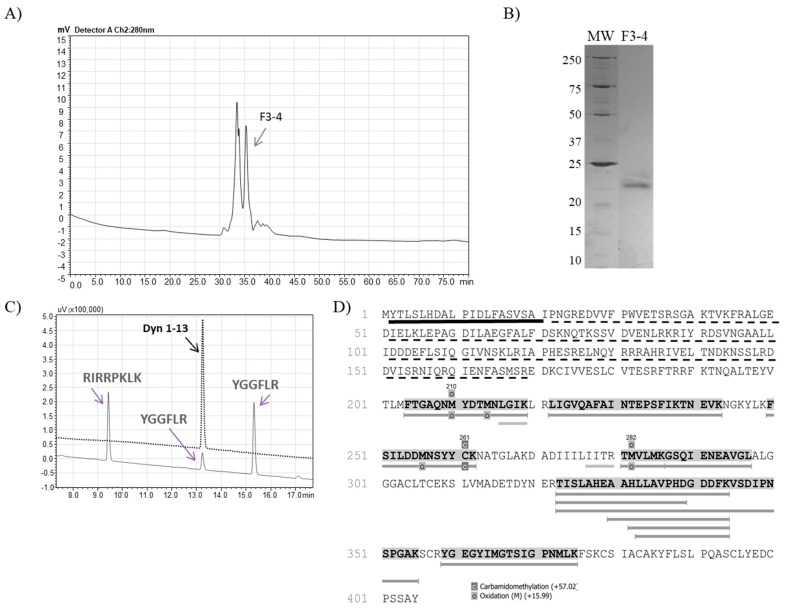
(**A**) Rechromatography on Shim-pack Diol-300 of fraction 3 from anion exchange chromatography. The arrow indicates the peak containing proteolytic activity on the FRET and Dyn A (1-13) substrates, with Leu-enkephalin releasing. (**B**) SDS-PAGE (12%) analysis of F3-4 (2 µg) under non-reduced conditions. (**C**) Proteolytic activity over Dyn A (1-13) by F3-4, hydrolysis of which was visualized on the C-18 RP-HPLC system (Shimadzu). (**D**) The mass spectrometry analysis identified F3-4 as metalloserrulase 3. The sequence underlined in black represent the signal peptide and the dotted lines represent the propeptide. Highlighted and underlined amino acids in grey represent the peptides sequenced by PEAKS Studio 8.0. The amino acids that are just underlined in grey are the ones that are found in *de novo* sequencing only.

Fractions F3 and F5 were then separately injected into a Diol-300 (Shim-pack) gel filtration column and the collected peaks were analyzed regarding hydrolysis upon dynorphin A (1-13) and FRET substrate. The resulting F3-4 subfraction from the second chromatographic step (Figure 2, panel A) was able to produce Leu-enkephalin from Dyn A (1-13) hydrolysis (Figure 2, panel C), and the electrophoretic profile demonstrates that fraction F3-4 had a single protein band with approximately 22 kDa (Figure 2, panel B). The mass spectrometry analysis of the F3-4 identified 14 unique peptides from the metalloserrulase 3 sequence, covering around 19% of the zymogen molecule and 57% of its mature form (Figure 2, panel D).

**Figure 3:**
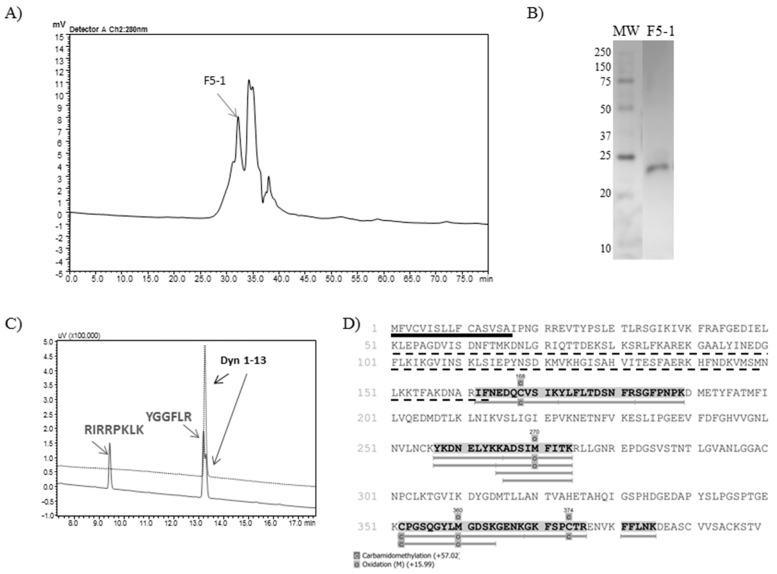
(**A**) Rechromatography on Shim-pack Diol-300 of fraction 5 from anion exchange chromatography. The arrow indicates the peak containing the active protease. (**B**) SDS-PAGE (12%) analysis of F5-1 (2 µg) under non-reduced conditions. (**C**) Proteolytic activity over Dyn A (1-13) by F5-1, hydrolysis of which was visualized on the C-18 RP-HPLC system (Shimadzu). (**D**) The mass spectrometry analysis identified F5-1 as metalloserrulase 4. The sequence underlined in black represents the signal peptide and the dotted line represents the propeptide region. Highlighted and underlined amino acids in grey represent the peptides sequenced by PEAKS Studio 8.0.

Regarding the subfractions resulting from the F5 gel filtration chromatographic step, F5-1 (Figure 3, panel A) was the only one which was able to cleave the substrate Abz-GFLRRV-EDDnp. Dynorphin A (1-13) was also cleaved by F5-1, with no Leu-enkephalin formation (Figure 3, panel C). Finally, the silver stained 12% polyacrylamide gel demonstrated a single protein band with 24 kDa (Figure 3, panel B). The mass spectrometry analysis of F5-1 identified the molecule as metalloserrulase 4, where 9 peptides covered around 17% of the total molecule and 32% of its mature form (Figure 3, panel D).

### 2.2. Bioinformatic Analysis

**Figure 4:**
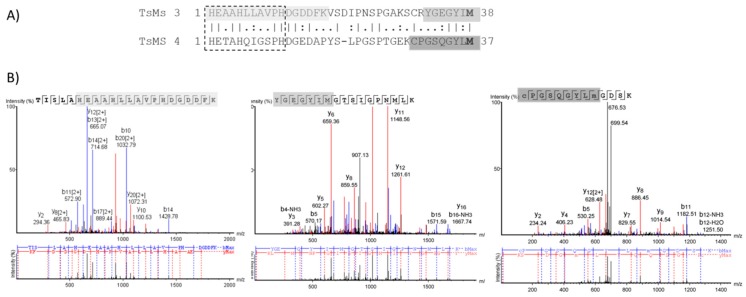
(**A**) Alignment of the extended zinc-binding sites of metalloserrulases 3 (TsMS 3) and 4 (TsMS 4). The sequence of the active site of metzincins, HEXXHXXG (A)/XXH is shown in the dashed box. The probable methionine residues (M) from the Met-turn region are in bold. (**B**) MS/MS spectra containing the peptides in the region of the extended active site. The highlighted sequenced fragments are also colored in Panel A.

Due to the different cleavage sites in Dyn A (1-13), the extended zinc-binding of metalloserrulases 3 and 4 were aligned using the BLAST tool (Figure 4). The alignment demonstrated 44.7% identity and 60.5% similarity between these two catalytic domains. Moreover, metalloserrulase 4 has the conserved glycine residue in the extended metallopeptidase motif HEXXHXXGXXH, which is a characteristic of the family called “metzincins”, while metalloserrulase 3 has an alanine instead of glycine in this motif. Furthermore, the probable position of Met-turn methionine for both metalloserrulases is shown in Figure 4, which was previously estimated by [16].

### 2.3. Comparative Analysis of Metalloserrulases Biochemical Parameters

**Figure 5:**
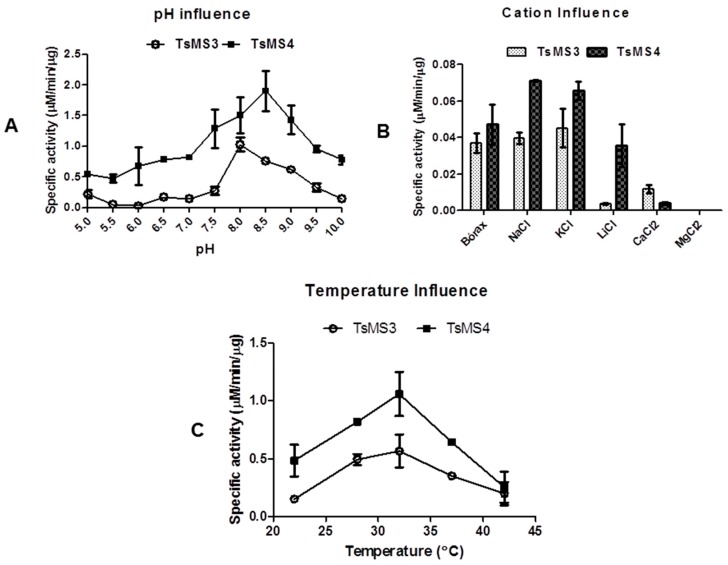
Initial biochemical characterization of the two metalloserrulases using the Abz-GFLRRV-EDDnp (5 μM) as substrate. (**A**) Evaluation of optimum pH from pH 5 to pH 10; (**B**) Influence of monovalent and divalent cations; (**C**) Thermo-stability study between 22 °C and 42 °C. All assays were made in triplicate.

Since TsMS 3 and TsMS 4 were purified for the first time, we aimed to perform an initial biochemical characterization of these enzymes in vitro. The catalytic activities of both metalloserrulases were evaluated in a pH range from 5.0 to 10.0 (Figure 5, panel A). For both proteases, a better proteolytic activity on alkaline rather than acid buffers was observed. The best activity was achieved at pH 8.0 for metalloserrulase 3, and at pH 8.5 for metalloserrulase 4. When Abz-GFLRRV-EDDnp was used as substrate, the specific activity of TsMS 4 is twice the activity of TsMS 3 in all studied pHs. Corroborating with this, the greater specific activity of TsMS 4 on the FRET substrate is also shown in results obtained in studies of cations influence and temperature effects (Figure 5, panel B and C, respectively).

The evaluation of the influence of monovalent (Li^+^, Na^+^, K^+^) and divalent cations (Mg^2+^, Ca^2+^) on the enzymatic activity of TsMS 3 and TsMS 4 was determined in fluorimeter with the addition of cations as chloride (50 mM) using borax buffer 50 mM, pH 8.5 at 37 °C, which was used as control (Figure 5, panel B). In general, metalloserrulases are positively affected by Na^+^ and K^+^, but not Li^+^. The negative influence of lithium was more drastic for TsMS 3 activity than for TsMS 4. On the other hand, the presence of divalent cations caused a decrease of TsMS 3 and TsMS 4 activities, but it was less expressive for metalloserrulase TsMS 3 in comparison to TsMS 4. Magnesium at the concentration used (50 mM) was able to completely inhibit the activity of both metalloserrulases.

The thermo-stability was also tested and, in general, metalloserrulases 3 and 4 behaved in the same way over temperature variations (Figure 5, panel C). Both had an optimum peak of activity at 32 °C, and were active at all temperatures in the tested range. At 42 °C, however, proteolytic activity of TsMS 3 and TsMS 4 decreased around 50%, indicating a possible denaturation of proteins.

### 2.4. Determination of Cleavage Sites

The cleavage point produced by metalloserrulase 3 and metalloserrulase 4 over the fluorescent substrate analogous to dynorphin A (1-13), Abz-GFLRRV-EDDnp, was determined, as described [29]. As expected, metalloserrulase 4 formed only a single cleavage point between Arg-Arg; for TsMS 3, two cleavage sites were obtained: between Arg-Arg and between Leu-Arg.

**Figure 6:**
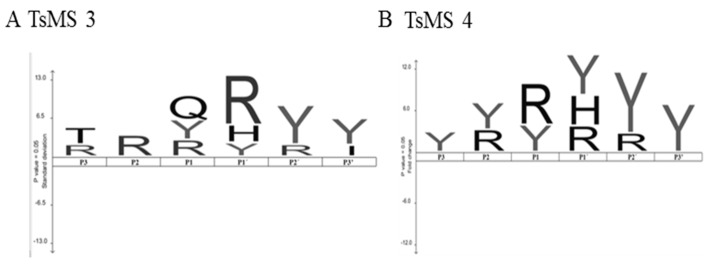
Preliminary study of primary specificities of metalloserrulase 3 and metalloserrulase 4 based on cleavage points on peptides belong to the neuropeptide Y family and dynorphin A (1-13), using the IceLogo software [31]. (**A**) IceLogo of amino acid position preference on substrates by metalloserrulase 3; and (**B**) by metalloserrulase 4.

The scissile bonds and specific activities were also determined, as described [21], for the NPY, PYY, PP and dynorphin A (1-13) hydrolyses produced by metalloserrulases 3 and 4 (Table 2), which allowed for the analysis of the primary specificity of these proteases (Figure 6).

The peptides were incubated with proteases and the specific activity was determined on RP-HPLC. The peaks corresponding to the hydrolysis products were analyzed on mass spectrometry to determine the cleavage sites (see experimental details in Section 5.4.3).

Both metalloserrulases cleaved the amidated C-terminal portion of the three peptides belonging to the neuropeptide Y family (Table 2). These cleavages lead to the inactivation of these neuropeptides, since the C-terminal part is responsible for binding with their receptors [30]. Table 2 shows that neuropeptide Y was the most susceptible substrate for the metalloserrulases tested, presenting a greater number of cleavage points (17 cleavage points for TsMS 4, and 14 for TsMS 3) when compared with the other substrates, which presented 10 hydrolysis sites or less. Some hydrolysis points are shared by both metalloserrulases (Table 2, grey enhancement), however, most fragments are unique to each peptidase, indicating different primary specificities for these enzymes. In terms of specific activity, both metalloproteases show high hydrolysis rates of Dyn A (1-13), followed by neuropeptide Y. In comparison, Dyn A (1-13) is most efficiently cleaved by TsMS 3 (1.54 µM/µg/min) than TsMS 4 (1.3 µM/µg/min). On the other hand, TsMS 4 have a higher contribution on the degradation of the neuropeptide Y than TsMS 3 (0.631 µM/µg/min and 0.433 µM/µg/min, respectively). According to the data obtained by the IceLogo software, the preference for arginine in the P1 position was significant for metalloserrulase 4, and in the P1′ position for TsMS 3. Moreover, metalloserrulase 3 may interact with a glutamine (Q) in the P1 position in addition to recognizing Arg and Tyr (Figure 6).

### 2.5. Kinetic Parameters Determination

The obtainment of the Michaelis–Menten (K*_m_*) and catalytic (k*_cat_*) constants for hydrolysis of Dyn A (1-13) analogous FRET substrates (Abz-GFLRRV-EDDnp, Abz-GFLRR-EDDnp, and Abz-FLRRV-EDDnp) by metalloserrulases 4 was performed with increasing substrate concentrations. All kinetics were fitted to the hyperbolic Michaelis–Menten rate equation and the results are shown in Table 3. TsMS 4 hydrolyzed all substrates at Arg-Arg bond. On the other hand, metalloserrulase 3 recognized the sequences in two peptide bonds (Leu-Arg and Arg-Arg) and, thus, it was not possible to determine the catalytic constants for the hydrolysis of these substrates (Supplementary Figure S2).

Among all substrates analyzed, the Abz-GFLRRV-EDDnp presented the lowest value of K*_m_* (16.2 μM), indicating that the largest sequence had the best interaction with TsMS 4. The C-terminal valine, which interacts with the S’2 subsite, was shown to be an important factor for TsMS 4 activity, since its catalytic efficiency on the Abz-GFLRR-EDDnp substrate was drastically reduced, both for presenting the lowest k*_cat_* value (170 s^−1^) and the highest K*_m_* value (36.6 μM) of all studied sequences. In the same way, the presence of glycine in P4 seems to help the substrate to bind in the protease catalytic pocket, as the K*_m_* value for the Abz-FLRRV-EDDnp (28.1 μM) is higher than the K*_m_* obtained for Abz-GFLRRV-EDDnp hydrolysis (16.2 μM). However, the presence of a glycine at the P4 position negatively affected the k*_cat_* value, which is about twice as low for the Abz-GFLRRV-EDDnp (492 s^−1^) when compared to the Abz-FLRRV-EDDnp substrate (1108 s^−1^). Based on the specificity constants (k*_cat_*/K*_m_*), the best substrate tested was the Abz-FLRRV-EDDnp (39.4 µM^−1^ s^−1^).

### 2.6. In Vitro Neutralization Assay of the Activity of Metalloserrulases by Commercial Antivenoms

**Figure 7:**
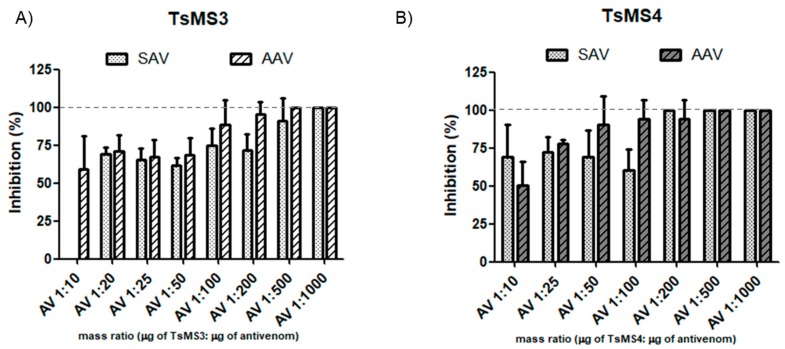
Neutralization assay of isolated proteases using the substrate Abz-FLRRV-EDDnp (**A**) Inhibition of TsMS 3 (88 ng) and (**B**) TsMS 4 activities (100 ng) in PBS buffer pH 7.4. Proteases and antivenoms were previously incubated without the substrate, for 30 min at 37 °C, in seven different proportions (protease mass/antivenom mass ratio): 1:10; 1:25; 1:50; 1:100; 1:200; 1:500 and 1:1000, and their hydrolysis rates were compared to the control (without antivenom). The results are the average of three independent experiments and expressed as percentage of the inhibition of each metalloserrulase activity. SAV = scorpion antivenom and AAV = arachnidic antivenom.

As shown in Figure 7, the in vitro neutralization assay demonstrated effectiveness even in low amounts of antivenom (venom/antivenom mass ratio of 1:20). For TsMS 3, the neutralization by the arachnidic antivenom (AAV) showed earlier inhibition than scorpion antivenom (SAV). In contrast, TsMS 4 was, in general, equally neutralized by both antivenoms. For both proteases, an increase in inhibition was observed with higher amounts of antivenoms, and the full neutralization was reached at 1:500 in all cases, except SAV for TsMS 4 (which was 1:200).

## 3. Discussion

The study of isolated toxins in animal venoms is an important step to identify functions of new molecules or even to better understand envenomation mechanisms. For proteases of the *Tityus serrulatus* venom, it may be understood that its action is associated only with post-translational events, since 80% of peptides identified in the venom are proteolytic products of endogenous toxins [22], or, in addition, that they may also be enzymes that could play important roles in the envenoming. To date, metalloserrulases have only been detected in the cDNA library of the *Tityus serrulatus* scorpion venom gland, selected according to homology with the proteases belonging to the M13 class of metalloproteases [16]. Although this approach represents important evidence of their presence in the venom, it is necessary to confirm whether metalloserrulases 3 and 4 are actually toxins of the *T. serrulatus* venom. This is the first report on the isolation and characterization of the proteolytic activities of these two active metalloserrulases that are present in the *T. serrulatus* venom, metalloserrulases 3 and 4, which surely will help on further studies with these enzymes.

Metalloserrulases 3 and 4 were purified to homogeneity by two chromatographic steps and their identities were confirmed by mass spectrometry analysis. The molecular masses predicted for the active forms of TsMS 3 and TsMS 4 are 22 kDa and 26 kDa [16], respectively. The molecular mass of the TsMS 3 is in agreement with the electrophoretic profile obtained with this metalloserrulase, which presents a protein band of approximately 22 kDa. On the other hand, the metalloserrulase 4 presented migration rate compatible with a protein with molecular mass of 24 kDa. Two facts may explain this divergence from 26 kDa to 24 kDa - the mature portion of the metalloserrulase 4 molecule has been estimated in in silico studies and may contain deviations, or the purified molecule underwent post-translational modifications and still exhibits catalytic activity. The primary structure of the metalloserrulase 4 shows a metzincins zinc-binding consensus sequence, **HE**TA**H**QI**G**SP**H**, which includes three protein ligands of the catalytic zinc and the general base/acid glutamate for catalysis. Interestingly, the metalloserrulase 3 has an alanine residue instead of glycine, **HE**AA**H**LL**A**VP**H**, and it is important to note that this unusual feature of the TsMS 3 was confirmed in the mass spectrometric analyzes presented in this study. Another characteristic common to metzincins is the presence of a Met-turn that is structurally and spatially conserved, and distanced by 6-53 amino acids from the third zinc-binding histidine in the different metzincin structures [32]. In accordance with previous results obtained with antarease [17], alignment studies of extended binding sites of TsMS 3 and TsMS 4 indicate the presence of conserved methionine residues, which are separated from the first histidine by connecting segments of 38 and 37, amino acids residues, respectively. Peptide fragments containing the Met residues were also sequenced on the two metalloserrulases in mass spectrometric analyzes performed.

The first biochemical analyses with TsMS 3 and TsMS 4 aimed to determine the optimum levels of three physical-chemical parameters (pH, temperature and cations effects), so that the obtained results could be used in the subsequent studies of their hydrolytic activities on neuropeptides. As previously determined for the venom of *T. serrulatus* [23], a pH of 8.5 was defined as optimal for metalloserrulase 4. The ideal pH for the metalloserrulase 3 activity was determined as 8.0, followed by 8.5, where the protease also showed a high level of proteolytic activity. Regarding thermo-stability studies of the molecules, in general both metalloserrulases behaved in the same way with temperature variations—probably due to the ectothermic nature of scorpions—and both metalloserrulases had an optimum activity peak at 32 °C. However, at 42 °C the proteolytic activity decreased by about 50% for both metalloserrulases, indicating a possible denaturation of the enzymes’ secondary and tertiary structures. Lastly, it is known that the presence of cations commonly interferes with the proteolytic activity, either enhancing or inactivating enzymes. It was possible to observe a positive influence of monovalent cations, especially sodium and potassium, and negative influence by divalent ions, such as magnesium and calcium. Both metalloserrulases were inhibited by divalent cations, which can act as competitors of the zinc at the catalytic site, causing its substitution and, thus, destabilizing the enzyme [33].

Although the use of a greater number of substrates for a more accurate primary specificity study would be best, the preliminary results presented here, using the cleavage points obtained on peptides belonging to the neuropeptide Y family and dynorphin A (1-13), provided relevant information on preferences of the metalloserrulases 3 and 4 for the hydrolysis of substrates. For both, a high preference was observed for the interaction of arginine and tyrosine residues with the S3–S3′ subsites, regardless of their positions. However, arginine residues are most frequently found in the P1 and P1′ positions on substrates hydrolyzed by TsMS 4 and TsMS 3, respectively. This fact may explain the exclusive Leu-enkephalin releasing (YGGFL) from the hydrolysis of dynorphin A (Y^1^G^2^G^3^F^4^L^5^R^6^R^7^I^8^R^9^P^10^K^11^L^12^K^13^) by TsMS 3, since the two cleavage sites, Leu^5^-Arg^6^ and Arg^6^-Arg^7^, were observed to have arginine residues in the P1′ position of the substrate. It is important to consider that the cleavage site between arginine residues is characteristic of processing proteases for the activation of precursor molecules [34], which could reinforce the suggestion of the actions of the metalloserrulases as post-translational agents in endogenous venom toxins, as previously described for a protease purified from the *Cupiennius salei* spider venom [22,35].

The metalloserrulases showed the same primary specificity for the Abz-G^1^F^2^L^3^R^4^R^5^V^6^-EDDnp hydrolysis already evidenced in the studies with dynorphin A (1-13), that is, TsMS 3 cleaved the substrate at two points (Leu^3^-Arg^4^ and Arg^4^-Arg^5^), whereas TsMS 4 was capable of hydrolyzing a single peptide bond (Arg^4^-Arg^5^). Considering that the FRET substrate is cleaved by TsMS 4 with a single-cleavage point, it was possible to perform a deeper substrate-specificity study. The best FRET substrate tested was Abz-FLRRV-EDDnp, and both the removal of valine from the C-terminus and the addition of a glycine residue in the N-terminus decreased TsMS 4 catalytic efficiency. The primary specificity results, although preliminary, are robust evidence of an extended binding site for both metalloserrulases to interact with substrates.

Metalloserrulases 3 and 4 were able to remove in vitro the carboxy amidation of NPY, PP and YY, which leads to the inactivation of the biological activities of these peptides [30]. The three peptides belonging to the neuropeptide Y family are widely distributed in the body and act through several subtypes of G-protein-coupled Y receptors [28]. Neuropeptide Y is the most abundant neuropeptide in the central and peripheral nervous systems in mammals and has been implicated in different activities ranging from the control of anxiety to angiogenesis and cardiovascular function [26,36]. PYY and PP are mainly contained in the pancreas and gastrointestinal mucosa and, in particular, the pancreatic polypeptide is related to diseases of the pancreas, such as pancreatitis [37]. The inactivation of Dyn A (1-13) and the release of Leu-enkephalin during the envenomation process may lead to unexpected consequences, as this neuropeptide may act in vivo on both opioid and non-opioid receptors, and can interact with potassium ion channels promoting indirect neurotoxicity [38,39]. Dynorphin A (1-13) is also released by immune cells locally during painful inflammation [25,40], which is a condition observed in in vivo studies with *Tityus serrulatus* venom [41]. In spite of the fact that there can be significant differences between in vivo and in vitro results, it is possible that these neuropeptides are targets for the metalloserrulases during the envenomation process. This hypothesis should be checked by further in vivo studies with isolated proteases.

Since the WHO’s recommendation in case of human accidents with venomous scorpions is immunotherapy, serum neutralization assays using commercial antivenoms were performed. Neutralization assays results demonstrated effective inhibitions of activities of both metallopeptidases when using the two commercial antivenoms produced by the Butantan Institute. Surprisingly, the activity of TsMS 3 was more efficiently inhibited by the lowest dose used of anti-arachnidic serum when compared to the same dose of anti-scorpion serum. To explain this result, we hypothesized that the other two spider venoms used to compose the immunization pool to obtain the AAV (*Phoneutria nigriventer*—21.5%—and *Loxosceles gaucho*—21.5%) may contain metalloproteases that share epitopes with the TsMS 3. Regarding the venom of *Loxosceles intermedia*, an astacin-like metalloprotease has been described, which presents a 21% similarity with metalloserrulase 3 [42]. Despite the satisfactory results obtained in the serum neutralization assays, and since the action of peptidases is usually rapid, it is important to consider that the immediate onset of immunotherapy after the accident is crucial for the treatment of the victims. Otherwise, cleavages of bioactive peptides, if they occur during envenomation, are initiated and trigger their physiological effects.

Recently our group demonstrated that the *T. serrulatus* venom proteases are indicative of the venom’s toxicity, since venom batches with lower proteolytic activities over the FRET substrate showed higher LD_50_ values, and therefore are less toxic in comparison to the batches with greater hydrolytic activities [43]. In this manner, metalloserrulases 3 and 4 may be important toxins from the venom, acting in the formation or inactivation of human neuropeptides, besides being responsible for the maturation of endogenous peptides [22].

## 4. Conclusions

In conclusion, this study describes the purification and characterization of two novel metallopeptidases from the *T. serrulatus* venom, acting upon human neuropeptides in in vitro studies. Since the TsV has neurotoxic effects, mainly attributed to molecules without enzymatic activity, it is possible that human neuropeptides degradation may also have an important role in the envenomation process.

## 5. Materials and Methods

### 5.1. Reagents

Dynorphin A (1-13), neuropeptide Y, peptide YY and pancreatic polypeptide were purchased from Sigma-Aldrich (St Louis, MO, USA). Acetonitrile and trifluoroacetic acid (TFA) were acquired from J.T. Baker. The fluorescent resonance energy transfer (FRET) substrates Abz-GFLRRV-EDDnp, Abz-FLRRV-EDDnp and Abz-GFLRR-EDDnp were kindly provided by Prof. Dr. Luiz Juliano Neto and Prof. Dr. Adriana Carmona, from the Department of Biophysics of UNIFESP-EPM.

### 5.2. Venoms and Antivenoms

The lyophilized venom of *Tityus serrulatus* (Batch no. 2146) was provided by the Venom Section of the Butantan Institute, SP, Brazil. The scorpion and the arachnidic antivenoms (SAV and AAV, respectively) were obtained from Hyperimmune Plasmas Processing Section, Butantan Institute, SP, Brazil. The SAV (batch no 0905104/A) and the AAV (batch no 0706121) contained protein concentrations of 8.43 g/dL and 15.4 g/dL, respectively. The antivenoms from the Butantan Institute are produced through the hyperimmunization of horses with a pool of *T. serrulatus* venom (100%) for SAV, and *T. serrulatus* (57%), *Phoneutria nigriventer* (21.5%) and *Loxosceles gaucho* (21.5%) venoms for AAV [44].

### 5.3. Purification of Metalloserrulases 3 and 4 from Tityus serrulatus Venom

#### 5.3.1. Chromatographic Steps

The lyophilized *Tityus serrulatus* venom (50 mg) was dissolved on 5 mL of 20 mM Tris, 20 mM NaCl, pH 8.2 buffer (final concentration 10 mg/mL). The TsV was first submitted to anion exchange chromatography in an HPLC system (Prominence, Shimadzu Co, Kyoto, Japan) using a Shim-Pack PA-DEAE column (20 mm × 100 mm) at 4 mL/min flow. The gradient used was 0–80% B in 80 min (buffer A containing 20 mM Tris, 20 mM NaCl, pH 8.2 and buffer B composed by buffer A with addition of NaCl 500 mM, pH 8.2). For salt removal, the fractions were submitted to a Millipore Amicon Ultra Centrifugal filter device (10 kDa MWCO, Amicon Co. Ltd., Bedford, MA, USA), following the manufacturer instructions. After the selection of active fractions (as described in Section 5.3.2 and Section 5.3.3), they were applied to a Shim-pack Diol-300 (7.9 mm × 50 cm) gel filtration column coupled to an HPLC system (Prominence, Shimadzu Co, Kyoto, Japan) and was eluted with 200 mM sodium sulfate, 10 mM sodium phosphate, pH 7.0 buffer at 0.5 mL/min flow rate in 80 min. Lastly, active subfractions were concentrated with a Millipore Amicon Ultra centrifugal filter device (10 kDa MWCO, Amicon Co. Ltd., Bedford, MA, USA), also using the manufacturer recommendations. For all chromatographic steps, UV detection was at 280 nm.

#### 5.3.2. Screening Using FRET Substrate

For endopeptidase activity screening, the Abz-GFLRRV-EDDnp substrate (5 μM) was incubated individually with each fraction collected from all chromatographic steps. Moreover, the relative inhibition of active fractions was determined using EDTA at 100 mM. The results using FRETs substrates were obtained on a fluorimeter (Victor 3, Perkin Elmer, MA, USA), adjusted for excitation and emission readings at 320 and 420 nm, respectively, and analyzed using the Grafit 5 software (Erithacus Software, West Sussex, UK). The temperature remained constant at 37 °C and one reading per minute was performed for 15 min, the plates being shaken before each measurement.

#### 5.3.3. Screening Using Human Neuropeptides

In parallel to the fluorimetric screening, the cleavage of Dyn A (1-13) (30 μM) was also verified, and each fraction collected was incubated with this peptide in a water bath at 37 °C for 90 min. The analysis was subsequently observed on HPLC reverse phase chromatography (Prominence, Shimadzu, Kyoto, Japan) using a Shim-pack Restek C-18 column (4.6 × 250 mm). Hydrolyses were analyzed by RP-HPLC, with 0.1% trifluoroacetic acid (TFA) in water as solvent A, and acetonitrile and solvent A (9:1) as solvent B. The used gradient was from 10% to 60% of solvent B in 20 min, with UV detection at 214 nm.

### 5.4. Characterization of Isolated Proteases

#### 5.4.1. SDS-PAGE—In Gel Digestion and Mass Spectrometry

The active fractions of each purification step were analyzed by 12% polyacrylamide gel electrophoresis (SDS-PAGE) [45]. Samples (2.0 µg) were solubilized in non-reducing sample buffer and silver-stained.

The purified proteases were subjected to an in-gel digestion with mass spectrometry grade trypsin from Sigma–Aldrich (St. Louis, MO, USA) [46]. The mixture was then desalted, concentrated and resuspended in 0.1% formic acid. Mass spectrometric analysis was performed by online liquid chromatography in an Easy-nLC Proxeon nanoHPLC system coupled to an LTQ-Orbitrap Velos (Thermo Fisher Scientific, Bremen, Germany) through a nanoelectrospray ion source. Raw data files were analyzed on Mascot Search Engine and on PEAKS Studio (version 8.0, Bioinformatics Solution, Waterloo, ON, Canada) against the library constructed with sequences deposited on UNIPROT/SwissProt, with a total of 554,567 sequences. A decoy database was also used to calculate false discovery rate (FDR) using the decoy-fusion method [47,48]. The search parameters were: trypsin cleavage specificity (max 1 missed cleavage); precursor mass tolerance set to 10 ppm; and a fragment ion mass tolerance of 0.5 Da. Regarding Post Translational Modifications (PTM), carbamidomethylation as fixed modification and oxidized methionine (Mþ15.994915 Da) and deamidation (NQ) as variable modification were considered. The peptide sequences that resulted from MS/MS were analyzed in Peaks DB and the matched peptides were filtered by FDR ≤ 1%, protein confidence score being −10lgP ≥ 62.

The deduced sequences of TsMS 3 and TsMS 4 transcripts from the gland of *T. serrulatus* [16] were aligned using NCBI Blast Tool.

#### 5.4.2. Biochemical Characterization: Effect of pH, Cation Concentration and Temperature

The Abz-GFLRRV-EDDnp substrate (5 µM) was used to determine the influence of pH, temperature and cations over the catalytic activity of metalloserrulase 3 (90 ng) and metalloserrulase 4 (150 ng). In all assays, the final volume used was 100 µL and hydrolyses were monitored on fluorimeter, as described on Section 5.3.2. Moreover, all results were obtained in triplicates and the mean and standard deviations (±SD) between the tests were determined. The specific activities (UF/min/µg) were obtained using the Grafit 5 software (Erithacus Software, West Sussex, UK).

For the study of pH influence over metalloserrulases activities, the following buffers were prepared, according to the recommendation of Stoll and Blanchard (1990): sodium phosphate (pH 5.0–7.5), borax (pH 7.6–9.2) and borax-NaOH (pH 9.2–10), all at the final concentration of 50 mM [49].

After the determination of the optimum pH, the influence of monovalent and divalent cations on both peptidases activities was studied with Borax pH 8.5, in the presence of several types of mono and divalent cations as their chlorides (Na^+^, K^+^, Li^+^, Mg^2+^, Ca^2+^), at final concentration of 50 mM. The rate of hydrolysis in the presence and absence of each ion was evaluated, identifying possible changes in TsMS 3 and TsMS 4 enzymatic activities.

The influence of temperature on metalloserrulases activities was also analyzed. For this, the activities of both peptidases were analyzed at temperatures between 22 °C and 42 °C, on 5 °C intervals, using the best buffer indicated based on the results of the previous experiments (Borax 50 mM, NaCl 50 mM, pH 8.5).

#### 5.4.3. Analysis of The Cleavage Sites

To determine the cleavage points of the Abz-GFLRRV-EDDnp substrate (5 µM) obtained by metalloserrulase 3 and metalloserrulase 4 activities, 150 ng of each protease were incubated in 50 mM borax buffer pH 8.5 for 15 min (final volume at 100 μL). Hydrolyses were monitored on fluorimeter Victor 3 (Perkin Elmer, MA, USA), as described in Section 5.3.2, and the cleavage was subsequently observed by RP-HPLC (same conditions as described on Section 5.3.3, with gradient modification varying from 30% to 80% of solvent B in 25 min). The peaks were collected manually and then the cleavage points of each sample were determined in a MALDI TOF/TOF mass spectrometer (Axima Performance, Shimadzu Co, Kyoto, Japan) and the scissile bonds were deduced from the sequences of the substrate fragments. One microliter of each sample was co-crystallized with a supersaturated solution of α-cyano-4-hydroxycinnamic acid matrix (50% acetonitrile/water/0.1% TFA, 50/49.9/0.1, *v*/*v*/*v*), deposited on the sampler and dried at room temperature. The samples were analyzed and the spectra were acquired using the linear positive mode.

For the neuropeptides cleavage points determination, metalloserrulases 3 and 4 (150 ng) were incubated with the natural substrates Dyn A (1-13) (30 µM), neuropeptide Y (23.4 µM), peptide YY (23.2 µM) and pancreatic polypeptide (22.7 µM), for 6 h at 37 °C in 50 mM Borax buffer 50 mM NaCl, pH 8.5 (final volume at 100 µL). The cleavages were observed on reverse phase chromatography C-18 (Section 5.3.3) and the hydrolysis products were manually collected to be analyzed by mass spectrometry (Section 5.4.1). The *de novo* peptide sequences were obtained by software PEAKS Studio (version 8.0, Bioinformatics Solution, Waterloo, ON, Canada), with the following parameters: no specificity of the enzyme; precursor mass tolerance of ±10 ppm and an ion fragment mass tolerance of ±0.5 Da; oxidized methionine (M +15.994915 Da) was defined as a variable modification. The identified peptides were classified according to their Average of Local Confidence (ALC) and those that had ALC > 80% were selected. The obtained cleavage points were, thus, utilized to study the primary specificity for substrate hydrolysis of TsMS 3 and TsMS 4. For this, amino acid corresponding to P3-P3′ positions [50] were analyzed on the IceLogo tool [31], allowing frequency visualization of amino acid residues in each studied position.

#### 5.4.4. Kinetic Parameters for The Hydrolysis of FRET Substrates by TsMS 4

Since TsMS 3 cleaved the FRETs substrates at two sites, these analyzes were performed only with TsMS 4. For determination of the Michaelis–Menten (K*_m_*) and catalytic (k*_cat_*) constants, sequential amounts of fluorescent substrate were used. The substrates analyzed were: Abz-GFLRRV-EDDnp, Abz-FLRRV-EDDnp and Abz-GFLRR-EDDnp, at concentrations of 2.5 μM, 5 μM, 10 μM, 15 μM, 25 μM, 50 μM and 100 μM. The buffer used for metalloserrulase 4 was borax pH 8.5. Enzymatic activities were monitored on fluorimeter in the same conditions described on Section 5.3.2 for 30 min. The standard hydrolysis conditions were strictly maintained for different substrates and the limit of substrate hydrolysis was 10% (initial rates of hydrolysis). The kinetic parameters were calculated by Michaelis–Menten equation using the Grafit 5 software (Erithacus Software, West Sussex, UK). For the k*_cat_* (s^−1^) calculation, the *maximum velocity* (*V_max_*) obtained in relative units of fluorescence per minute (UF/min) were converted to μM of substrate cleaved per minute (μM/min). The conversion was based on the total hydrolysis of 1 μM Abz-FLRRV-EDDnp (1440 UF = 1 μM). All experiments were performed in triplicates and represented as mean ± SD.

### 5.5. Sera Neutralization Assays

The potential of commercial antivenoms to neutralize metalloserrulases activities was evaluated by in vitro assay. For this, both metalloserrulases were pre-incubated with commercial antivenoms (scorpion or arachnidic antivenoms produced by the Butantan Institute, as described in Section 5.2) for 30 min in PBS buffer, pH 7.4. The concentrations (venom: antivenom ratio, in μg) tested were: 1:10; 1:25; 1:50; 1:100; 1:200; 1:500 and 1:1000. After the pre-incubation period, the substrate Abz-FLRRV-EDDnp (5 μM) was added and the neutralization determined in Victor 3 fluorimeter, as described in Section 5.3.2. All experiments were performed in triplicates and represented as mean ± SD.

## Figures and Tables

**Table 1:** Summary of purification of metalloserrulases TsMS 3 and TsMS 4 from the *Tityus serrulatus* venom.

Step	Fraction	Volume (µL)	Total Protein (µg)	Activity (units)	Total Activity (units/µL)	Specific Activity (units/µg)	Purification Factor	Yield (%)	EDTA Inhibition (%)
Venom	TsV	1000	30,000	90	90,000	3000	1	100	100
DEAE	F3	1000	0.12	13.1	13,100	109,167	36.39	14.6	100
	F5	1000	0.139	20.9	20,873	150,165	50.06	23.2	100
GF	F3-4	1000	0.024	8.9	8900	370,833	123.61	9.9	100
	F5-1	1000	0.071	12.7	12,729	179,276	59.76	14.1	100

The fluorescent substrate Abz-GFLRRV-EDDnp (5 µM) and the EDTA (100 mM) were used to screen the proteolytic activity. DEAE = anion exchange chromatography; GF = Gel filtration chromatography.

**Table 2:** Specific activities and cleavage points produced by metalloserrulases 3 (↑) and 4 (↓) in the neuropeptide Y (NPY), peptide YY (PYY), polypeptide pancreatic (PP) and Dyn A (1-13) (DYN). The cleavage sites shared by both peptidases are highlighted in light grey.

Peptide	Specific Activity (µM/µg/min)		Scissile Bonds
	TsMS 3	TsMS 4	
NPY	0.433	0.631	YPSKP↑D↓NPGED↓↑A↑PAEDM↓A↓↑R↓↑Y↓↑Y↓↑S↓A↓L↓↑R↓↑H↓↑Y↑I↓N↓↑LIT↓R↓↑Q↓↑RY-NH_2_
PYY	0.359	0.420	YPIKPEAPGED↓ASPEEL↓N↓R↓YYASL↓↑R↓HY↓L↓NLVT↑R↓Q↓RY-NH_2_
PP	0.347	0.430	APLEPMYP↓G↑D↑Y↑A↑TH↑E↑Q↑RAQ↑YETQL↓↑R↑R↓↑Y↑YPIKPE↓↑PRY-NH_2_
DYN	1.539	1.303	YGGFL↑R↓↑RIRPKLK

**Table 3:** Determination of catalytic constants for FRETs substrate hydrolysis by metalloserrulase 4.

	Substrate							Catalytic Constants		
	P4	P3	P2	P1	P1′	P2′		K*_m_* (µM)	k*_cat_* (s^−1^)	k*_cat_*/K*_m_* (µM^−1^·s^−1^)
Abz	G	F	L	R	R	V	EDDnp	16.2 ± 4.3	491.7 ± 52.4	30.4 ± 8.1
Abz	G	F	L	R	R	-	EDDnp	36.6 ± 5.8	170.8 ± 8.4	4.7 ± 0.7
Abz	-	F	L	R	R	V	EDDnp	28.1 ± 2.6	1108.3 ± 33.2	39.4 ± 3.64

## Supplementary Materials

The following are available online at https://www.mdpi.com/2072-6651/11/4/194/s1, Figure S1: Dynorphin A (1-13), 30 μM, cleavage profiles by (**A**) Fraction F3 and (**B**) Fraction F5. Each fraction was incubated with this peptide in a water bath at 37 °C for 90 min. Hydrolyses were visualized in the C-18 RP-HPLC system (Shimadzu) with UV detection at 214 nm. (**C**) Each individual peak corresponded to hydrolysis products were collected and analyzed by LC-MS to determine the monoisotopic mass and, therefore, its sequence, Figure S2: Chromatographic profiles of the fluorescent substrate Abz-GFLRRV-EDDnp hydrolysis by metalloserrulase 3 (line 1, grey) and metalloserrulase 4 (line 2, black) in comparison to the integral peptide (line 3, dotted). The confirmation of each peak content was performed by mass spectrometry analysis.
