# Supplementary material for: Purification and Biochemical Characterization of TsMS 3 and TsMS 4: Neuropeptide-Degrading Metallopeptidases in the Tityus serrulatus Venom
Source: Toxins (Basel). 2019 Mar 31;11(4):194. doi: 10.3390/toxins11040194 (PMC6520902; doi:10.3390/toxins11040194)
Supplement: Supplementary file 1 [file toxins-11-00194-s001.pdf]

Supplementary Materials: Purification and Biochemical Characterization of TsMS 3 and TsMS 4: Neuropeptide-Degrading Metallopeptidases in The *Tityus serrulatus* Venom

Daniela Cajado-Carvalho, Cristiane Castilho Fernandes da Silva, Roberto Tadashi Kodama, Douglas Oscar Ceolin Mariano, Daniel Carvalho Pimenta, Bruno Duzzi, Alexandre Kazuo Kuniyoshi and Fernanda Vieira Portaro

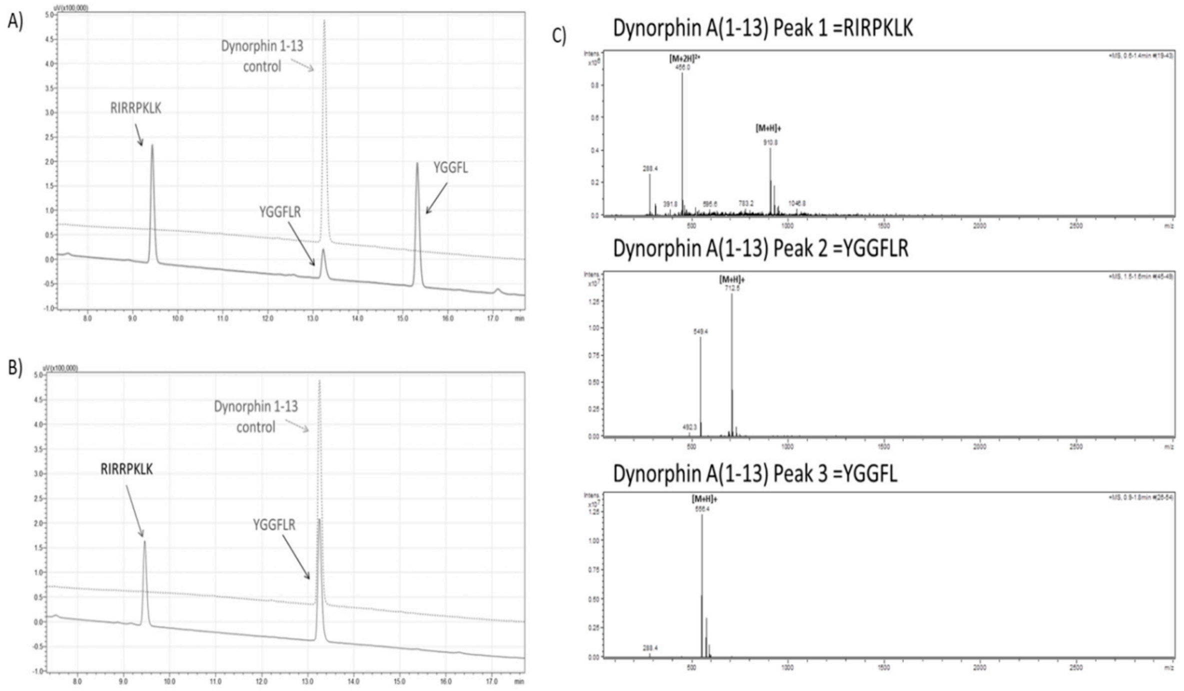

Figure S1. Profile of Dynorphin A (1-13), 30 μM, cleavaged by (**A**) Fraction F3 and (**B**) Fraction F5. Each fraction was incubated with this peptide in a water bath at 37 °C for 90 minutes. Hydrolyses were visualized using a C-18 RP-HPLC system (Shimadzu) with UV detection at 214 nm. (**C**) Each individual peak corresponded to hydrolysis products were collected and analyzed by LC-MS to determine the monoisotopic mass and, therefore, its sequence.

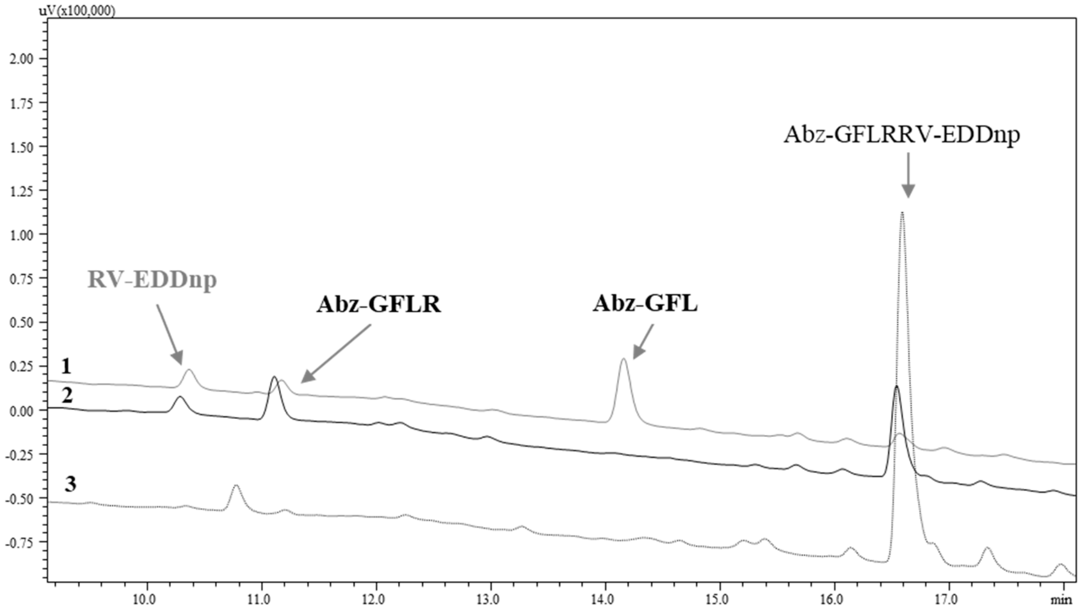

Figure S2. Chromatographic profiles of the fluorescent substrate Abz-GFLRRV-EDDnp hydrolysis by metalloserrulase 3 (line 1, grey) and metalloserrulase 4 (line 2, black) in comparison to the integral peptide (line 3, dotted). The confirmation of each peak content was performed by mass spectrometry analysis.
